# The real-time shadow detection of the PV module by computer vision based on histogram matching and gamma transformation method

**DOI:** 10.1038/s41598-024-71710-x

**Published:** 2024-09-18

**Authors:** Xinyi Liu, Haonan Xia, Ke Li, Yinghui Lu, Shanshan Lv, Qinghe Zhao, Weixian Song, Lishu Wang

**Affiliations:** 1 Electrical Engineering and Information College, Northeast Agricultural University, Harbin, China; 2 Division of Arts, Shenzhen University, Shenzhen, China

**Keywords:** Digital image processing, Photovoltaic module, Real-time, Shadow detection, Photovoltaics, Electrical and electronic engineering

## Abstract

Solar energy plays an important role in renewable energy generation, with the advantages of low pollution, easy installation, and relatively easy access. However, photovoltaic (PV) modules are susceptible to cause localized shading from external factors such as leaves in the canopy, surrounding buildings, etc., which would affect power generation efficiency and even pose safety risks. Existing methods cannot perform well in real-time conditions. This paper proposes a real-time shading monitoring method for the PV module based on computer vision. The gamma transform and histogram matching were adopted to enhance key features and adjust the global gamut strength distribution in the image of the PV module; then the gray-level slicing method finished the segmentation to detect the shadow from the video. All processing can be realized in the real-time monitor camera and the detection results can be displayed on the HMI in PC with high efficiency and low cost. According to tests in the practical complex environment, the method can have enough detection performance and high real-time performance with an accuracy of 0.98, and the F0.5 and F2 values are 0.87 and 0.85, respectively. The metrics of the proposed method are higher than those of the existing Canny detection method, the Random Forest detection method, and the CNN detection method. In addition, the average time required by the proposed method to process a frame is 0.721 s. In addition, the average time required by the method to process an image frame is 0.721 s, which has good real-time performance.

## Introduction

Solar energy plays an important role in renewable energy generation, with the advantages of low pollution, easy installation, and relatively easy access. The average annual solar radiation reaching the earth's surface is 4 × 1015 MW, equivalent to 3.6 × 105 billion standard coal equivalents^1^. Photovoltaic (PV) module efficiency is affected by factors such as solar irradiance, module surface temperature, and the presence of shading^2^. As the sun's position changes, PV systems are subject to localized shading from vegetation and surrounding buildings, which can result in uneven light distribution. In addition, the above event causes problems such as hot spots in PV panels, and multiple peaks in P–V characteristics, et al.^3,4^.

To avoid the negative effects of shading on power generation, large PV arrays with many modules must be manually inspected periodically or monitored by PV power generation system parameters such as voltage and current, and more.

Regarding electrical characteristics, Hariharan et al. proposed to classify the operating state of PV arrays into normal operation, partial shading, and fault state by calculating the output current, voltage, irradiance, and PV array energy loss^5^. Davies et al*.* proposed a PV array shading detection method based on fill factor (FF) and voltage inflection point. The method detects shading by calculating the FF of the PV array and combining the voltage inflection point, which appears on the I-V characteristic curve under local shading conditions. The voltage inflection point calculates the shading area^6^.

Traditional methods for detecting local shading in PV arrays, which are based on mathematical models and PV system parameters, are affected by factors such as the connection method of PV modules and the lifetime of PV modules. They cannot accurately detect the shading position and area of PV modules. These two methods are less efficient and cannot be monitored in real-time. It can seriously affect the power generation efficiency and pose a safety hazard. Therefore, it is necessary to improve the shadow detection method of PV array and realize intelligent operation and maintenance.

Machine vision is an essential method in shadow detection in a PV array by a high-resolution camera in a fixed location or unmanned aerial vehicle (UAV) in the air^7^. It can realize real-time monitoring the whole day at a lower cost, and there will be no additional manual operation before necessary maintenance.

In the field of digital image recognition of PV module shadow, Ye et al. proposed a PV module shadow recognition algorithm based on stack line smoothing and local threshold segmentation. They also proposed a simplified simulation method for the output characteristics of PV arrays considering local shading conditions^8^. Wang et al*.* proposed the use of Canny edge detection and other techniques to detect shadow areas, which is more accurate than image morphology processing methods. This method mainly uses image processing techniques and does not require information such as the electrical characteristics of PV arrays, which are difficult to measure^9^. Liu et al*.* proposed a PV module shadow region detection method based on digital image processing. The method uses a Hough transform to detect the component boundary lines and an improved threshold segmentation method to extract the shadow region^10^.

Image enhancement algorithms include frequency-domain and spatial-domain methods. The frequency-domain method treats the image as a two-dimensional signal, and enhances the signal by Fourier transform, which is commonly used, such as low-pass filtering and Gaussian filtering. In terms of gamma transformation, Huang et al. proposed adaptive gamma correction using weighted distributions, which can automatically improve image contrast by using a smoothing curve. Furthermore, they employed temporal information to reduce the computational time for several image frames of a video sequence^11^. Sahnoun et al. compared MRI contrast enhancement techniques based on conventional gamma correction (TGC) and adaptive gamma correction (AGC). It is concluded that TGC is superior to AGC in terms of image quality, brightness and structure preservation, however, image details are more conserved by AGC method by performing better value of entropy^12^. Ganguly et al*.* fused mathematical morphology with adaptive gamma correction to improve image visibility, and it is experimentally verified that the proposed method is able to eliminate the halo artifacts in the restored image^13^. In terms of histogram applications, Fazli et al*.* proposed an adaptive histogram equalization (HE) method that uses HE in multiple local window size regions, and it emphasizes local contrast, rather than overall contrast^14^. wang et al*.* proposed a method to accurately estimate γ parameters by analyzing the histogram gap distribution features and zero-value features of gamma-corrected images, and the experimental results show that the proposed method not only outperforms the existing methods in terms of parameter estimation accuracy, but also has good robustness to JPEG images with different quality factors before transformation^15^.

The methods of correction of illumination unevenness of the image are mainly divided into two categories: correction methods with reference and correction methods without reference. The former requires corrections with reference to a sample image, e.g. histogram matching, while the latter can correct the image directly, e.g. gamma transformation, algorithms based on Retinex theory, and morphological filtering. To better correct the geometric distortion, highlight the important details in the image, and further improve the accuracy of shadow detection, this paper synthesizes the reference correction and nonreference correction methods, and proposes a real-time shadow monitoring method for the PV module based on histogram matching and gamma transformation.

This paper proposes a real-time shadow monitoring method for the PV module based on histogram matching. First, the light is adjusted by gamma transformation and histogram matching to enhance the contrast, and then real-time shadow monitoring is achieved by grayscale layering and other methods. Through field experiments, the proposal method can successfully identify PV module shadows in real-time videos under different lighting conditions. The average accuracy rate is 0.98, and the lowest accuracy rate is 0.80. The currently commonly used recognition method based on Canny edge detection has an average accuracy of 0.95 and a minimum accuracy of 0.70. It can be seen that our method has better robustness, higher accuracy, and enough real-time performance.

This method has a significant reference value for shadow monitoring of large PV arrays. It provides new technical means for intelligent and accurate operation and maintenance of PV systems. We believe this work can potentially monitor the shadow of PV arrays in real-time, and we look forward to further research to extend it to more application scenarios.

The rest of this paper has the following structure.

Section "Material and method" describes the methods proposed in this manuscript. Among it, Section "Shaded PV module image features" analyzes the image characteristics of normal and partially shaded PV modules. Section "Real-time shadow monitoring method for PV module" describes the three main algorithms used in this method. Section "Experiment design" presents the experiments conducted to verify the feasibility of the method proposed in this paper. Section "Evaluation metrics" presents the evaluation metrics of the feasibility of the technique. Section "Results" presents the experimental results of this paper's method. Section "Conclusions" concludes and highlights potential avenues for future research and development.

## Material and method

### Shaded PV module image features

**Fig. 1:**
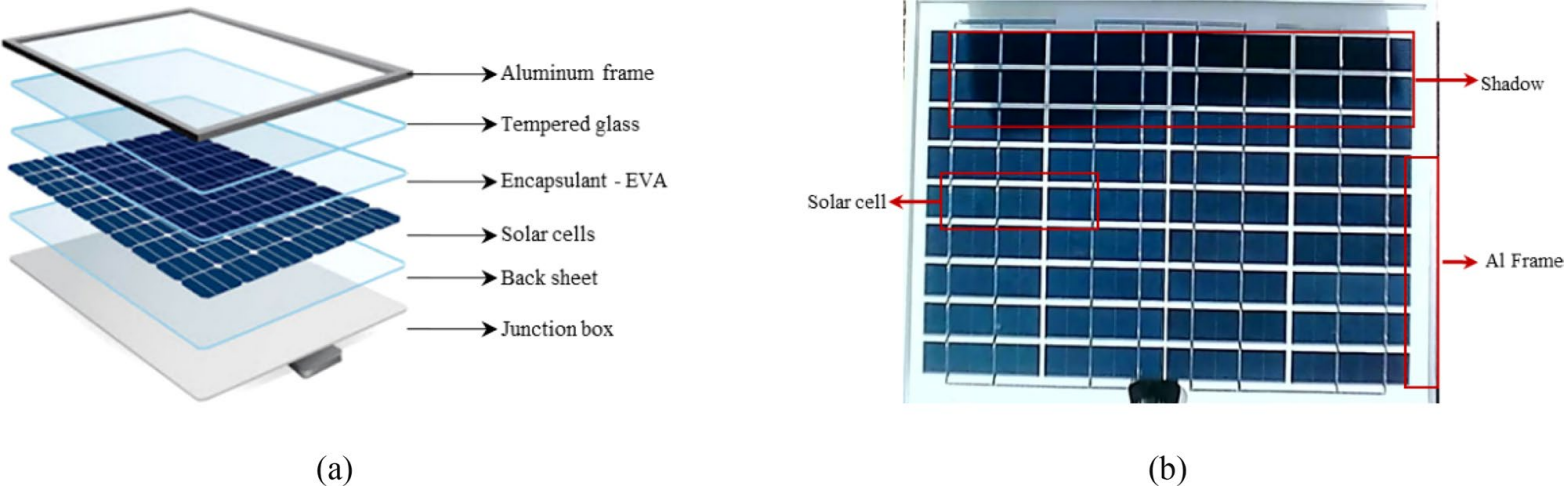
PV module introduction.

Typically, PV cells are composed of a high-transmittance glass cover, a dark crystalline silicon cell, a conductive silver paste, and an insulating back sheet as shown in Fig. 1a ^16^. The glass cover provides robust protection and light transmission for PV cells. The silver paste is used for busbars and contacts as an electrically conductive material to generate electricity in the silicon layer using solar radiation. As shown in Fig. 1b part, depending on the type of silicon wafer and anti-reflective coating used, PV modules usually appear dark such as blue or black color. The module frame is generally constructed from a silver-white alloy material, which tends to appear brighter in images. Under conditions of shading partially, the color of the shaded cell region is darker than that of the illuminated cell region. Furthermore, as the irradiance in the shaded region decreases, the color of the shaded cell region becomes progressively darker.

Grayscale histograms are commonly used to segment foreground and background in digital images and to segment the shadows of PV panels; the histogram can be used to select the threshold more accurately and facilitate segmentation. A grayscale histogram is a function of gray level and describes how often each gray level occurs in an image^17–19^. Thus, the peak of the histogram indicates that the gray level is the highest in the image.

**Fig. 2:**
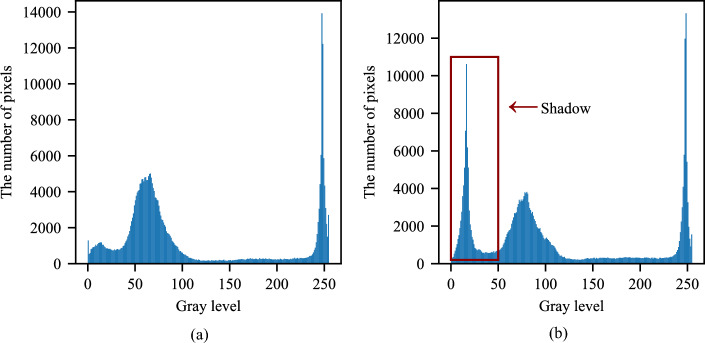
Grayscale histograms of unshaded and shaded PV modules.

As shown in Fig. 2, the image has significant differences between the normal and shaded parts. The x-axis is the gray level, and the y-axis is the number of pixels that appear in the gray level. The shaded part of PV cells appears darker in color, which results in lower gray levels of the shaded area. So, there is a significant wave in the gray scale from 0 to 50, which is the shadow area^20^. As shown in Fig. 2a, the gray level of the silicon cell that is not obscured by shadows ranges from 50 to 100. Gray levels around 250 represent the color of the white frame and silver paste.

As a basic tool, a grayscale histogram can show the color difference between the shadow and the rest, which can be used as a basis for shadow recognition. However, there are still the following difficulties in shadow detection.

#### Changes in light

Weak lighting conditions result in more color disparity but lower brightness in the shadow area, making it susceptible to noise interference. In intense light, the minimal color difference between the shaded and normal parts of the module surface makes them difficult to distinguish.

#### Shadows on PV back sheet gaps

**Fig. 3:**
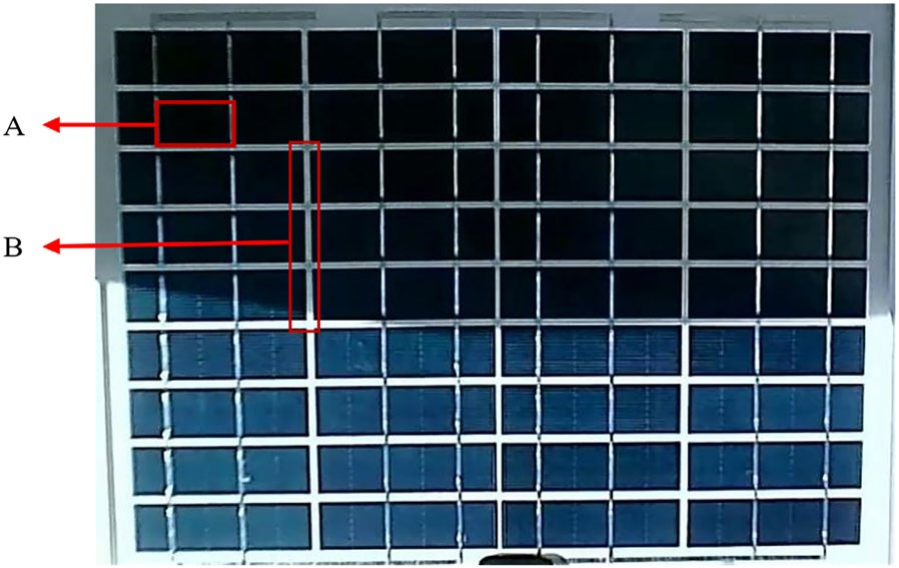
Shadows on PV Back Sheet Gaps.

Shadows on the PV back sheet gaps should be ignored, as the degree of shadowing does not affect the power generation efficiency of the PV module. As shown in Fig. 3, A denotes the shadows that affect the power generation efficiency, i.e., the shadows that need to be identified. B denotes the shadows on the PV back sheet gaps, which must be ignored in the shadow identification process.

#### Noise interference in images

Cameras inevitably generate noise, such as Gaussian noise, pretzel noise, etc., when capturing images. These noises affect image quality and are not conducive to shadow detection.

#### The irrelevant background interference

Since fixed cameras monitoring PV arrays need to cover many PV modules, the video images will inevitably contain information about the environment around the modules.

**Fig. 4:**
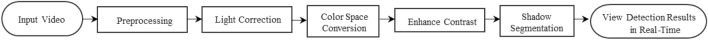
Flowchart of this shadow detection method.

This paper proposes a method for real-time monitoring of shadows on the surface of a PV module. As shown in Fig. 4, gamma transformation is used to adjust the light, histogram matching is used to enhance the image contrast, and grayscale slicing is used to detect the shadows based on the color features of the PV module and shadows.

### Real-time shadow monitoring method for PV module

In this paper, we propose a real-time shadow detection method for the PV, which realizes the shadow detection in the video by gamma transformation and histogram matching, et al*.* It has high accuracy and real-time performance.

About the difficulties in detecting the shadows of PV modules mentioned in the previous section, the proposal methods adopt the following measures. adjust the global gamut strength distribution.

**Fig. 5:**
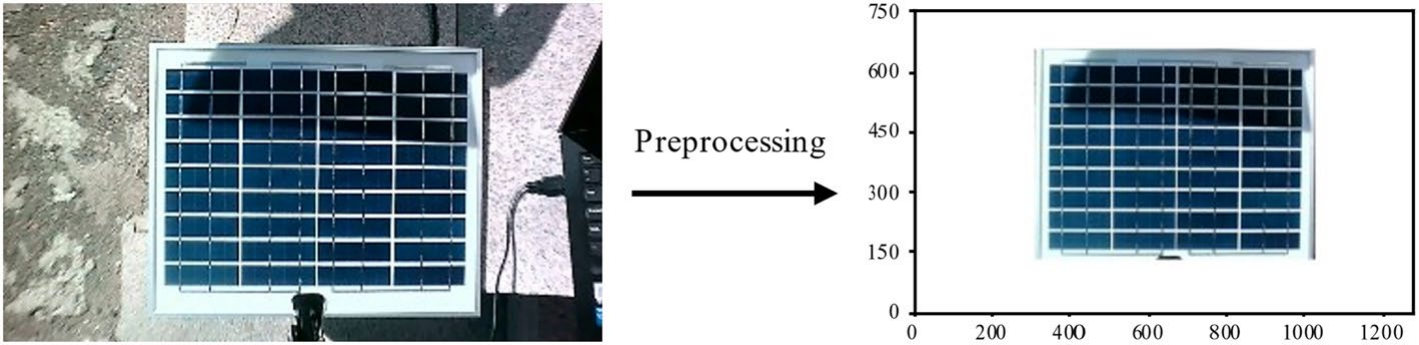
Image preprocessing.

To address the problem of illumination variation, this study implements gamma transformation and histogram matching to enhance the image and calibrate the overall gamut strength distribution^16–19^. The detection of shadows in the gaps of the back sheets can vary depending on the specific gray levels used because the back sheets are white and have a higher gray level than the shaded cells when they are shaded. This paper implements median filtering and Gaussian filtering to suppress image noise for improved detection accuracy^21,22^. To eliminate the background interference in the captured image, in this paper, we perform the preprocessing work before performing the shadow detection in the video. Based on the image coordinates, the PV components are classified as valid and retained, and the rest are invalid and displayed in white. Figure 5 shows the original image and the preprocessed output.

**Fig. 6:**
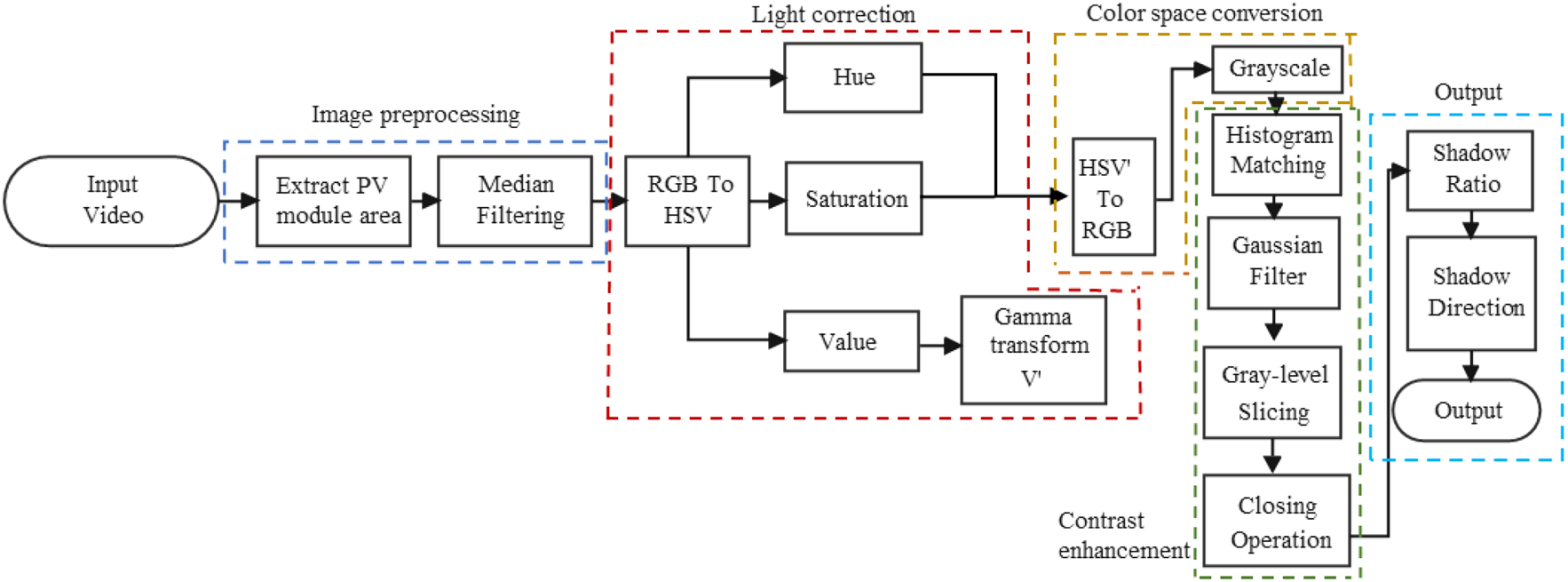
Flowchart of the shadow detection method proposal.

The real-time shadow monitoring method for PV module consists of preprocessing, the global gamut strength distribution, contrast enhancement, color space conversion, shadow segmentation, and output results. The methodology flowchart is shown in Fig. 6. And the specific steps are as follows.

1. The input video is preprocessed to capture and retain the PV module region based on the coordinates, and the remaining background portion is processed in white to prevent the environment from interfering with the shadow detection. Get the processed RGB image f (x, y).
2. Perform median filtering on f (x, y) while preserving the edge details of the image to remove noise and obtain g (x, y) ^23^.
3. Convert g (x, y) to HSV color space and extract the hue (H), saturation (S), and luminance (V) values. This process aims to maintain color consistency between the luminance-corrected image and the original image.
4. Perform a gamma transformation on the extracted V component to correct the light and obtain the brightness component V'^24^.
5. The S and H components extracted in step (3) are combined with V' to form a color image. In this paper, the HSV is converted to the RGB color space, and then grayscale is applied to obtain I (x, y) to retain more color information.
6. Perform histogram matching of I (x, y) to obtain q (x, y) by matching the image's histogram to be detected with the template's histogram to improve the image contrast^25^.
7. The image is filtered with a Gaussian low pass filter to eliminate high-frequency noise to obtain h (x, y)^26^.
8. Perform gray-level slicing on h (x, y), representing the gray value of the foreground area as ***white*** and the background area as ***black*** to produce a binary image k (x, y).
9. After performing gray-level slicing, a morphological closure operation is carried out on k (x, y) to derive m (x, y). Due to narrow discontinuities and thin gullies in the image, it becomes feasible to smooth out the contours and remove small voids.
10. Calculate the effect of shading and PV module orientation on power generation efficiency. Firstly, calculate the number of pixels in the f (x, y) function that cover all cell areas, i.e., the effective power generation area. Then, determine the black pixel percentage and location by calculating the number of black pixels in the m (x, y) function and using this value.

This paper's key to the method is the gamma transformation in the light correction module, the histogram matching in the contrast enhancement module, and the grayscale slicing in the shadow segmentation module. These three algorithms will be analyzed in detail below.

#### Gamma transformation

Gamma transformation enhances image sharpness, and aids shadow separation by nonlinearly transforming image brightness to improve light and detail.

The formula for the gamma transformation is defined by Eq. (1), where *s* is the output gray level after the correction, *r* is the input gray level corresponding to the initial image brightness, while c and γ are positive values^27^.

1 $$s={cr}^{\gamma }$$

**Fig. 7:**
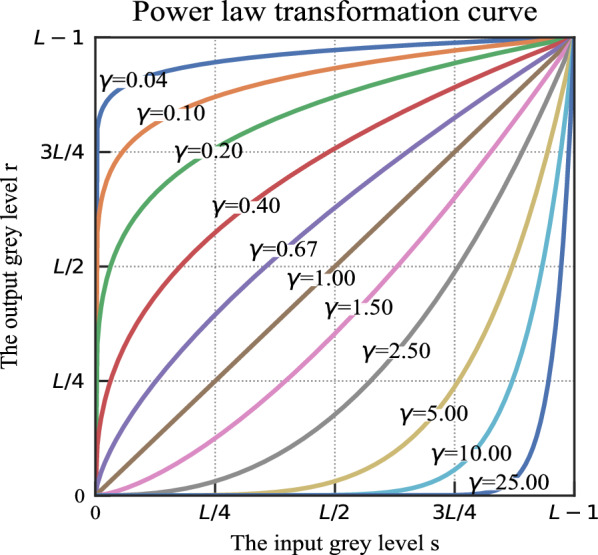
Gamma transformation curve.

Figure 7 shows the relationship between s and s for various γ values when c = 1. The x-axis represents the input gray level, whereas the y-axis represents the output gray level. The parameter γ is a parameter that controls the direction and strength of the transformation, and determines the type of mapping between the input image and the output image, i.e., whether low or high grayscale regions are enhanced. When γ = 1, a linear transformation takes place. When γ < 1, the low gray values are mapped to a broader range of output values, increasing the contrast between dark features and low gray areas in the image.

When γ > 1, the narrower range of high grayscale values is mapped to a broader range of output values. This process restores details in bright areas and enhances contrast in high-grayscale areas^28^.

Equation (2) calculates the number of distinct gray levels, denoted as L in Fig. 7, typically equating to a power of 2.

2 $$L={2}^{k}$$

The gamma transformation causes a portion of the pixel-level dynamic range of the image to be compressed and another portion to be stretched, and the functions have different concavities when γ > 1 or r < 1. Therefore, the transformed histogram will have new zeros and peaks, the gamma transformation will change not only the luminance, but also the ratio of red, green, and blue^29^.

The gamma transformation, as a nonlinear transform, can both resolve overexposed or too dark images and adjust the overall detail of the image. If the shadow part of the PV module itself is darker in colr, the gamma transformation will improve the contrast and then improve the detection of shadow with details. Considering real-time detection with balanced of not only computation but also speed of operation, methods such as adaptive gamma transform are not chosen, but a reasonable γ-value is determined based on half of the dataset, i.e. 2400 min of video.

**Fig. 8:**
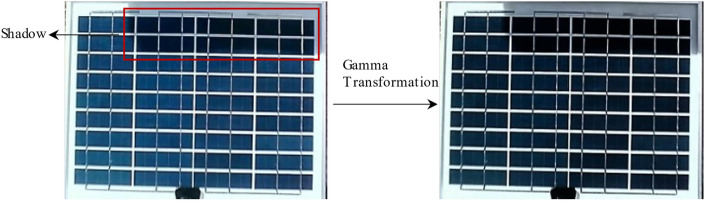
Gamma transformation effect diagram.

In this research, γ was configured with values of 0.5 to enhance the contrast of dark images to improve the visibility of details in shadow regions. The steps are (1) convert color space from RGB to HSV;(2) keep the hue H and saturation S components unchanged (3) perform gamma transformation on the luminance component V with γ = 0.5 to obtain the transformed luminance component V2. Combine it with the H and S components and convert it to RGB format. Figure 8 shows the effect of gamma transformation, where the shaded area is shown in the red box and the gamma transformed image is shown on the right.

**Fig. 9:**
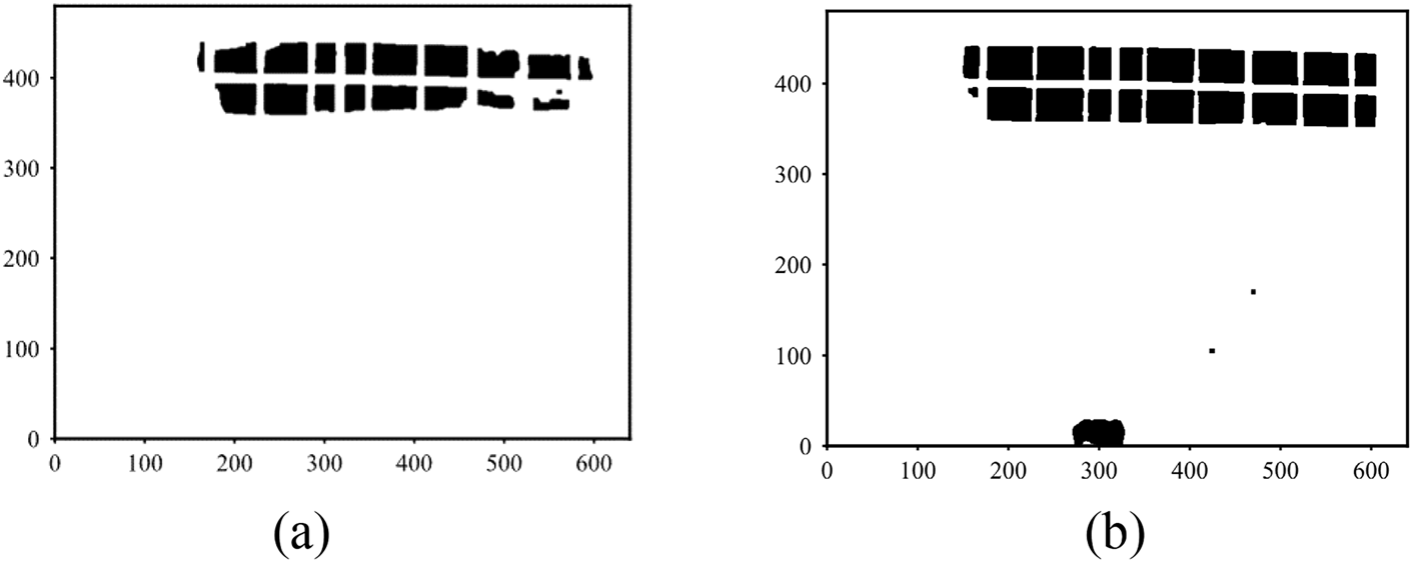
Comparison of shadow detection results.

Figure 9a shows the image obtained after gamma transformation through steps (5) to (8) in the sequence of this method. In contrast, Fig. 9b shows the shadow segmentation outcome without applying gamma transformation. The gamma transformation processed image significantly improves the precision of details within the shadow region, resulting in a more accurate shadow recognition.

#### Histogram matching

Histogram Matching improves image contrast by transforming the original grayscale histogram into the desired shape using a grayscale mapping function that changes the grayscale distribution of the image into a specific histogram interval. As shown in Eq. (3), the grayscale value of each point of the original image (input image) is mapped from r to s. Equation (4) maps the grayscale value of each end of the output image from z to v, where both *r* and *z* are greater than or equal to 0 and less than or equal to 1^30^.

3 $$s=T\left(r\right)={\int }_{0}^{r}{p}_{r}\left(r\right)dr$$

4 $$v=G\left(r\right)={\int }_{0}^{z}{p}_{z}\left(z\right)dz$$

5 $$z={G}^{-1}\left(v\right)$$

where *s* is the mapping of grayscale levels in the input image; *r* is the grayscale level in the input image; *p*_*r*_*(r)* is the continuous probability density function of the original image; *v* is the mapping of grayscale levels in the output image; *z* is the grayscale level in the output image, and *p*_*z*_*(z)* is the specified probability density function that the output image is expected to have in this paper^31^

s and v have the same distribution, i.e., T(r) = G(z), since both Eq. (3) and (4) are homogenizing transformations. From Eq. (5), it can be introduced that z satisfies Eq. (6), i.e., s is a mapping of z, and z is precisely the value expected in this paper when Eq. (6) is executed one by one on all pixels to obtain the corresponding elements in the output image^28,32,33^.

6 $$z={G}^{-1}\left[T\left(r\right)\right]={G}^{-1}\left(s\right)$$

Histogram matching, as a referenced image correction method, can map the histogram of the image recognized to a well-contrasted histogram of a template image, even if its gray levels have a specified probability density. The histograms of the shadows and the background will be significantly different after the transformation, which enhances the contrast between the raw image and transformed for easier distinguishment from the background in the histogram.

**Fig. 10:**
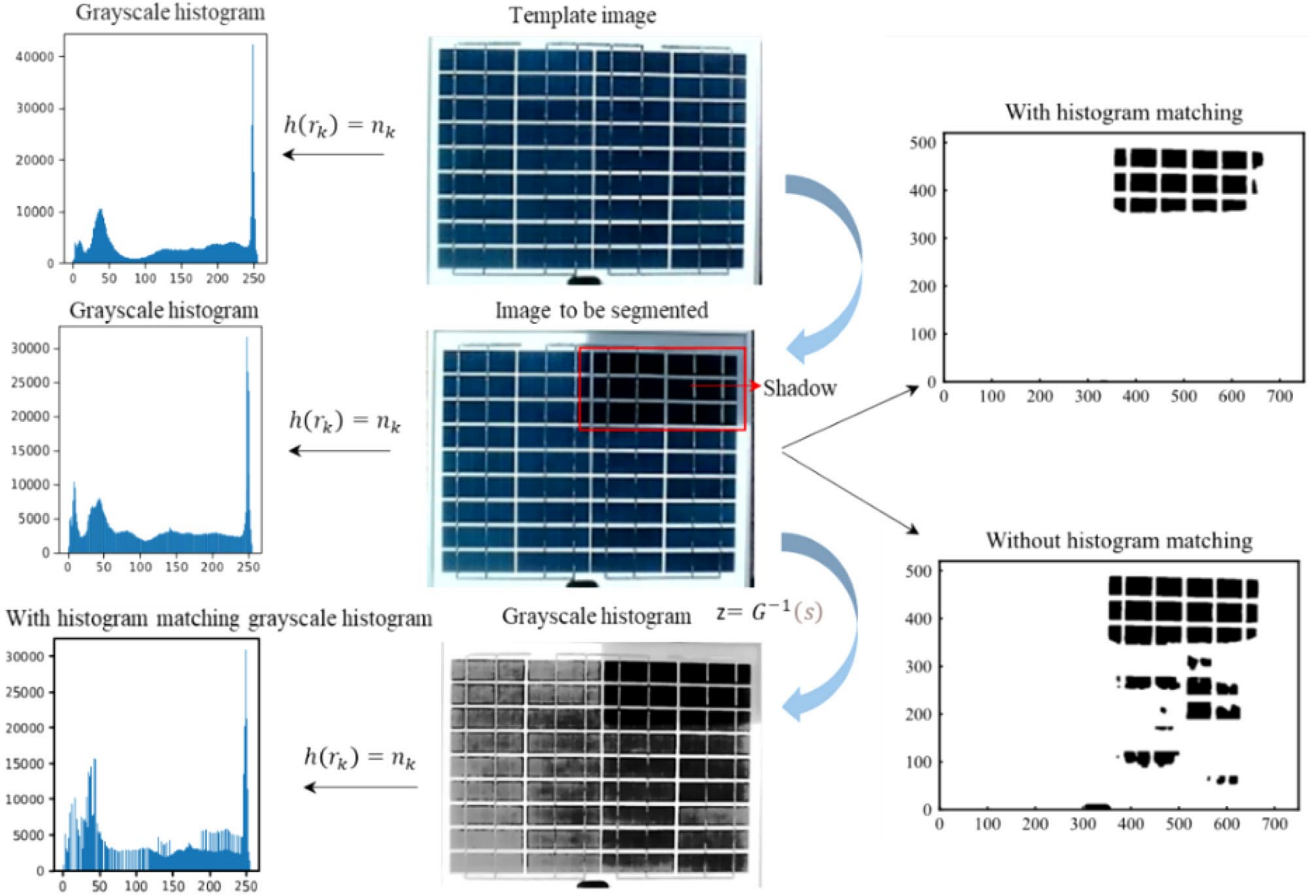
Visualizing the process and effects of histogram matching on shadow detection.

Figure 10 shows the performance and procedure of histogram matching on shadow detection. The left column of Fig. 10 shows the histograms of the target image, the image to be recognized, and the grayscale image after histogram matching, respectively, and it can be seen that the processed histogram has two obvious peaks, which makes it easier to segment the shadows. Figure 10 The middle columns are the template image, the image to be detected, and the grayscale image, respectively. The right column shows the recognition effect of the image with and without histogram matching, and it can be seen that the recognition method of adding histogram matching is more accurate for PV module shadow segmentation.

#### Gray-level slicing

Gray-level slicing is a segmented linear transformation function that enhances features by emphasizing the brightness of specific gray levels in an image to extract the target image.

**Fig. 11:**
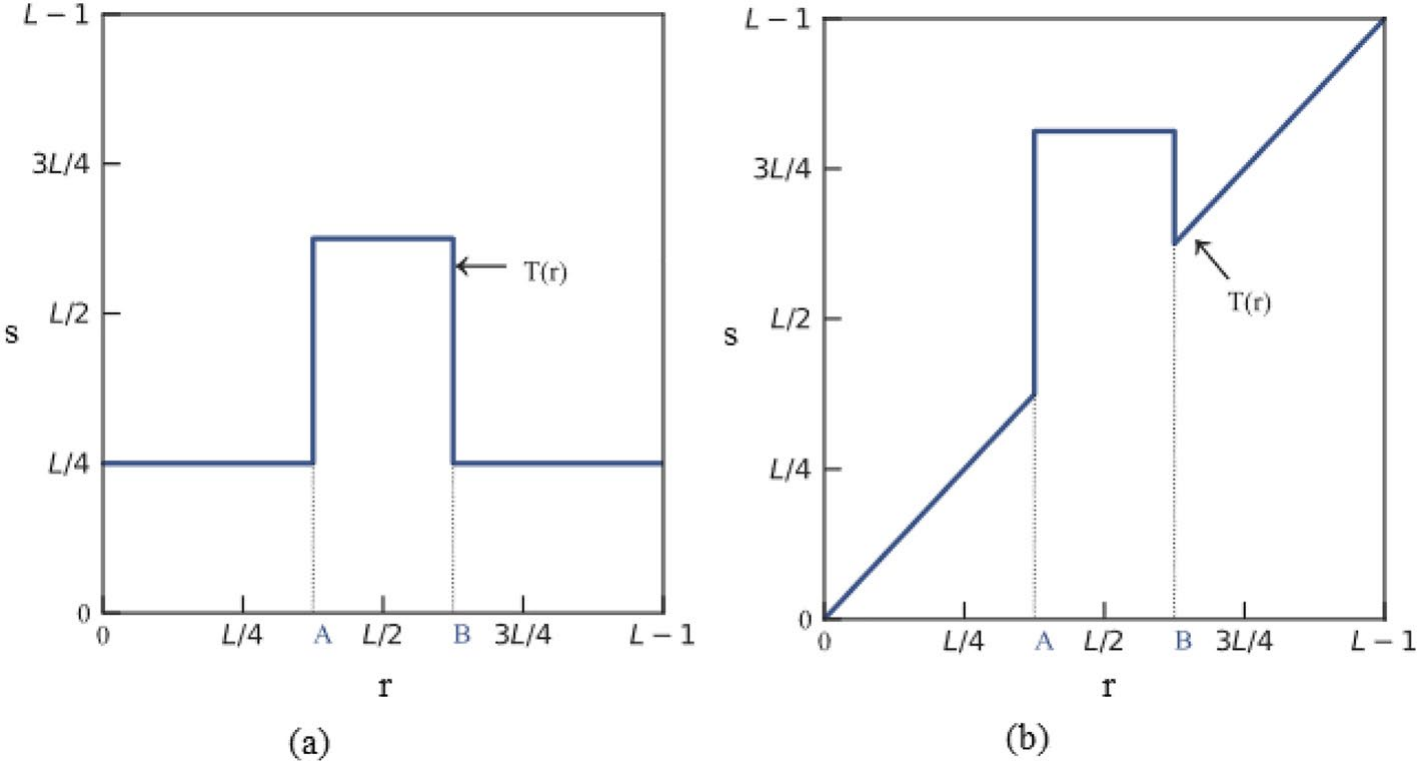
Gray-level slicing.

There are two methods of implementation. The first involves obtaining a binarized image with gray values within the range of interest being depicted as one value. In contrast, values outside of the field are displayed in another color. As illustrated in Fig. 11a, the grayscale values within the range of [A, B] are elevated and mapped as T(r), and the remaining grayscale values are reduced to lower levels. The second method makes the grayscale of the region of interest brighter or darker while leaving the grayscale of the other areas unchanged. As shown in Fig. 11b, the grayscale in the [A, B] region is emphasized, while all other grayscales remain unchanged^34^.

In this paper, we perform gray-level slicing as described in Eq. (7), and based on the gray-level histogram and color characteristics of the shaded PV module, a gray-level threshold of 15, denoted as G_xy_, where *G* is the changed grayscale value. This selection is based on the fact that the shaded area of the PV module has a darker color and lower than G_xy_ are depicted as ***black*** values, while areas with a higher value are depicted as ***white***^35–37^.

7 $$G = \begin{cases} 0, & G(i,j) \leq G_{xy} \\255, & G(i,j) > G_{xy}\end{cases}\ $$

**Fig. 12:**
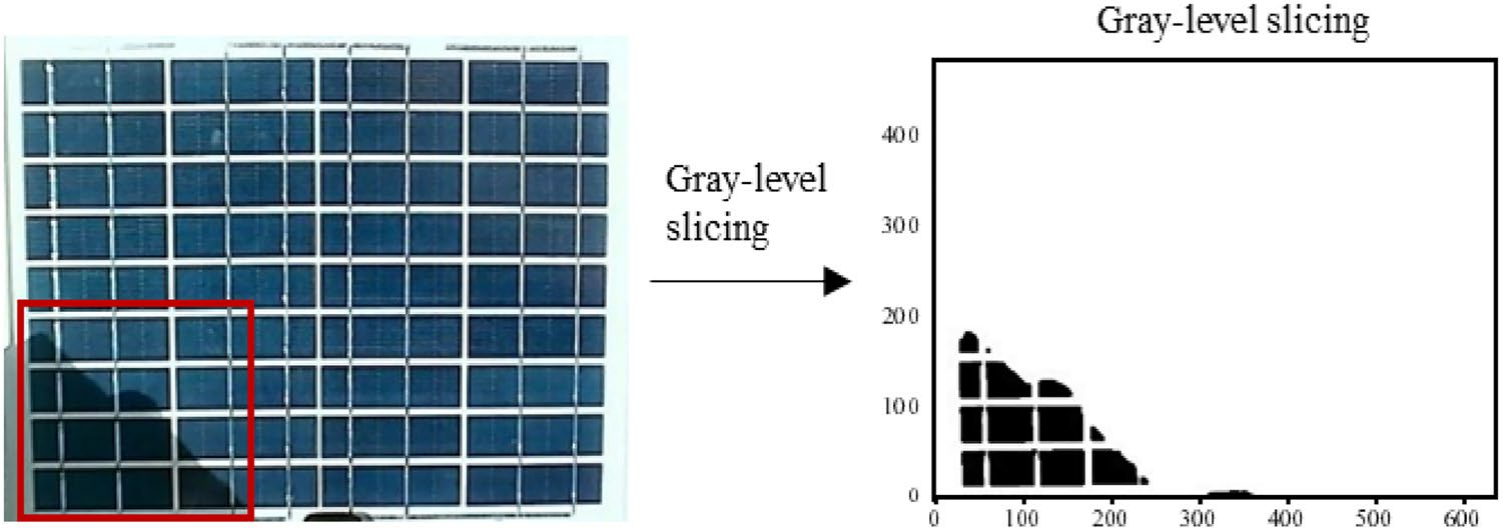
Gray-level slicing recognition results.

The localized shading component image to be identified is presented on the left in Fig. 12, with the shaded portion of the component surface in the red box. The figure on the right displays the recognition effect obtained from gray-level slicing processing used for shadow segmentation. This effect represents the final outcome of the method. The foreground, i.e., shaded areas, is mapped to black, and the background, i.e., nonshaded areas, is mapped to white^38,39^.

### Experiment design

A fixed real-time monitor for a PV array would cover not only PV modules, but also information about the environment around the modules in the video. To simulate the actual working environment better, the validation experiment in this paper designed as a fixed-position camera, with the environment around the PV module. In addition, this paper verifies the feasibility of the method for real-time monitoring of the shadow of a single PV module.

**Fig. 13:**
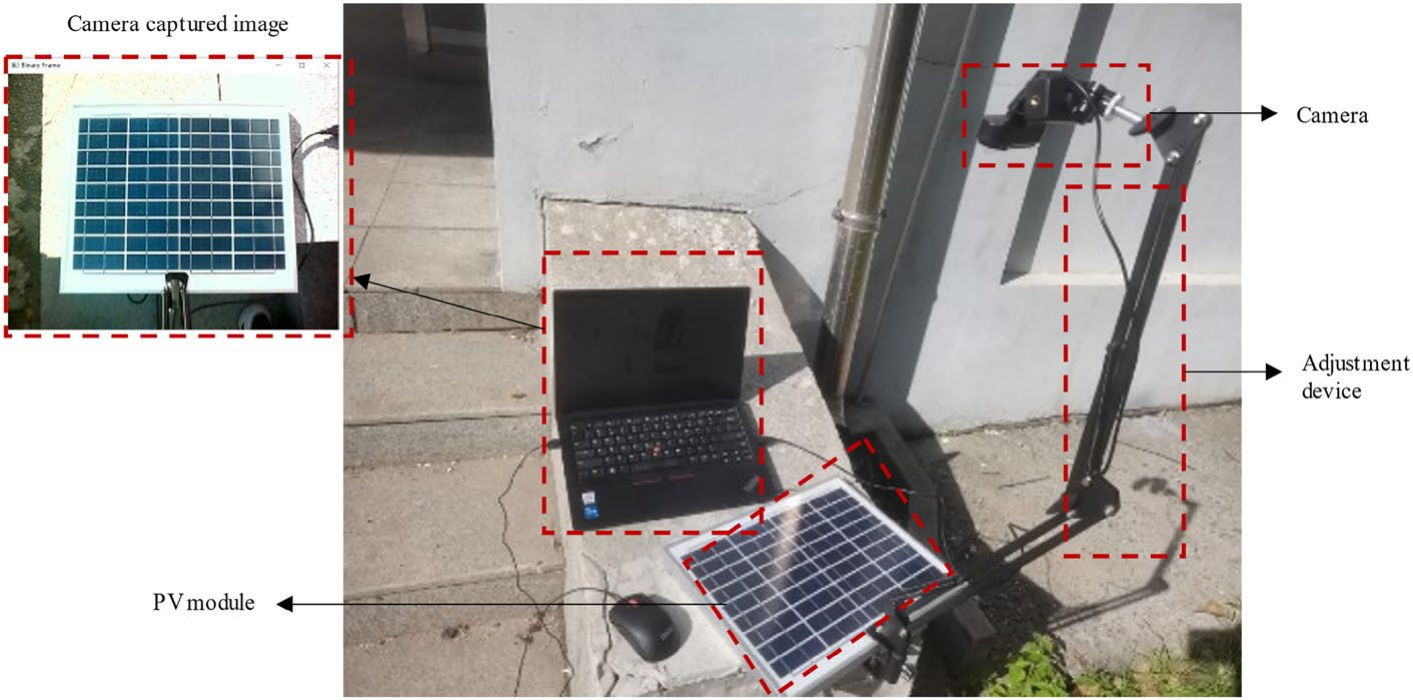
Experimental setup.

The image capture device shown in Fig. 13 consists of a mechanical bracket, a camera, and an adjusting device. The upper left corner of Fig. 13 shows the camera image. The alloy cantilever bracket is adjusted by spring tension and supports 180-degree folding and 360-degree rotation; the camera is fixed to frame with a frame rate of 30FPS; and the adjustment device adopts a multi-stage rotation mechanism that realizes 360-degree adjustments of the camera in the horizontal and vertical directions. The PV module comprises 36 solar cells of 220*770 mm, arranged in 4 columns and 9 rows, pasted on the white back sheet, and covered with tempered glass. In this paper, the camera is installed at a fixed angle to capture module images to simulate PV module working conditions as closely as possible.

The validation experiment was set in the small size PV modules, which would perform similarly in larger ones for their similar image features in color, shape and etc. with only differences of size. The color of the PV module surface affects the threshold selection of the gray level slicing module in this method. The color of the module surface depends on the color of the crystalline silicon cell wafers that make up the module, dark blue is one of the most commonly used colors for crystalline silicon cells. Therefore, only the dark blue small PV module is used as the data collection object in this paper.

**Fig. 14:**
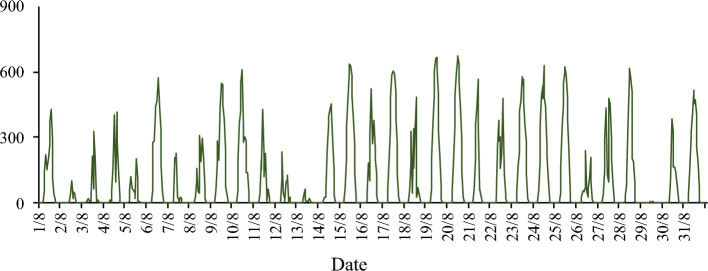
Direct solar radiation in August 2023.

To study the performance of the shadow detection method of the PV module by Computer Vision under different lighting conditions. This article is held on the campus of Northeast Agricultural University in Harbin, China (45°44′26″N, 126°43′03″E) from 1 to 31 August 2023 at 08:00–09:00, 12:00–13:00, 15:00–16:00, recording PV module video. The variation of direct solar radiation over time during the period is shown in Fig. 14.

The data collection is shown in detail in Table 1. A total of 90 videos of 60 min each were collected, giving a total of 5400 min of video data. In this paper, the videos are classified into two main categories based on the amount of radiation: radiation greater than 300 W/m^2^ is as sunny category, and radiation less than 300 W/m^2^ is considered cloudy category^1^. The video length is 4815 min for sunny days, 164 min for cloudy days and 421 min for rainy days and non-compliant videos. During this period, single and multiple cells were partially occluded, and the total occlusion time accounted for 86% of the whole recorded video, and the length of time when shading percentage exceeds 50% is 2003 min.

**Table 1:** Data set collection conditions.

Shadow generation conditions	Sunny (direct solar radiation > 300 W/m^2^)		Cloudy (direct solar radiation < 300 W/m^2^)	
	Shadow percentage < 50%	Shadow percentage > 50%	Shadow percentage < 50%	Shadow percentage > 50%
Number of videos recorded (unit: min)	2461	1678	115	28

In total, 4815 min of valid videos were collected at a resolution of 1280 × 720 pixels, capturing different lighting conditions and occlusion levels. Finally, 2400 min of video from the sample set were meticulously analyzed to determine various parameters, such as γ = 0.5 in gamma transformation, template image in histogram matching, Gxy = 15 in gray scale hierarchy and loss function in random forest model, and input image in CNN model. The remaining 2415 videos form a test set to evaluate the performance of this method.

### Evaluation metrics

The shadow detection of a PV module can be regarded as a binary classification problem, i.e., shadows are foreground, and nonshadow are backgrounds. This paper uses the following indicators to evaluate the performance of the methodology.

Masking is a data processing technique that controls the visibility of certain bits of data. By superimposing actual and predicted images. TP in the mask means true positive, FN means false negative, TN means true negative, and FP means false positive. The mask clearly shows the results of the shadow detection and indicates the method's accuracy.

Accuracy (ACC) metric can reflect the detection effectiveness of the methods generally and directly. This paper takes ACC as the main evaluation and its calculation as follows Eq. (8)^40^:

8 $$ACC=\frac{1}{{n}_{pixel}}{\sum }_{i=1}^{{n}_{pixel}}I\left(\widehat{y}={y}_{true}\right)$$

where $\widehat{y}$ is the predicted value corresponding to the true value y_true_ and n_sample_ is the number of samples in the dataset. However, while ACC can measure the method's overall performance, it is too macroscopic in its evaluation power, so we apply F-beta to get a perspective.

The F-beta score is the weighted harmonic mean of precision and recall, reaching its optimal value at 1 and its worst value at 0. The beta parameter represents the ratio of recall importance to precision importance. beta > 1 gives more weight to recall, while beta < 1 favors precision. For example, beta = 2 makes recall twice as important as precision, while beta = 0.5 does the opposite. Asymptotically, beta—>  + inf considers only recall, and beta—> 0 only precision. The calculation of F-beta is as follows Eq. (9):

9 $${F}_{\beta }=\left(1+{\beta }^{2}\right)\times \frac{precision\times recall}{{\beta }^{2}\times precision+recall}$$

where *precision* is the ratio tp/(tp + fp) and the *recall* is the ratio tp/(tp + fn).

The precision is intuitively the ability of the method not to label as positive a sample that is negative and the recall is intuitively the ability of the classifier to find all the positive samples. The precision and recall calculation are as follows Eqs. (10) and (11):

10 $$precision=\frac{TP}{TP+FP}$$

11 $$recall=\frac{TP}{TP+FN}$$

In this paper, TN denotes a pixel that is itself nonshaded and successfully predicted to be nonshaded; TP denotes a pixel that is itself shaded and successfully predicted to be shaded; FN is a pixel that is itself shaded but not perceived as nonshaded; and FP denotes a pixel that is itself nonshaded but mistakenly perceived as shaded. The more TP, the more accurate the model is in identifying shadows; the more TN, the more accurate the model is in identifying nonshaded; the smaller the FN, the more complete the model's recognition of shadows; the smaller the FP, the higher the accuracy of the model in identifying nonshaded.

## Results

In this paper, three groups of ablation experiments and three comparison groups are selected, to better evaluate the shadow detection performance of the model, where 1) to 3) are ablation experiments and 4) to 7) are comparison groups.

1. Change the gamma transform parameter γ from less than $1$ to $1.3$ in the method of this paper and keep the other steps unchanged.
2. Remove the gamma transform from the method in this paper and keep the other steps the same.
3. Remove the histogram matching from the method in this paper and keep the other steps the same.
4. Canny operator based shadow detection method in literature^9^: first, the grayscale image is binarized according to the Canny threshold, then the edges are detected and segmented, and the edges connected by breakpoints are filled to obtain the local shadow region.
5. In literature^41^, Ji et al. proposed the use of multi-threshold segmentation method calculates 10 thresholds, and the smallest threshold is used as the threshold for segmenting the local shadow region^41^.
6. In literature ^42^, Wang et al. proposed a random forest classifier with 50 decision trees, each with a maximum depth of 5, is used to calculate the intensity of shadows and edge features on a scale of 1–5^42^.
7. In literature^43^, Xia et al. proposed the use of convolutional neural network (CNN) to detect shadows with ReLU and Sigmoid activation functions, input size of three-channel images and output of masked images of the same size for determining the difference between shadows and other images^43^:.

The hardware environment that the analysis depends on is shown in Table 2 below and the compile environment is based on Python 3.9 in Windows 10 LTSC. To ensure reproducibility in all the experimental and analysis results, all random seeds involved in this paper are set as 615.

**Table 2:** Environment and tools for analysis and modeling in paper.

	Compute environment	Analysis tools
CPU	Intel®Core™i7-5500U(2.0 GHz)	Pandas 2.1.0, NumPy 1.26.3,
RAM	16 GB DDR4 2666 MHz	Scikit-learn1.3.0, CV2 4.8.0,
Operating system	Windows 10 LTSC	Scipy 1.11.2, TensorFlow 2.13.0,
Random seed	615	Scikit-Image 0.21.0,Keras 2.13.1

**Fig. 15:**
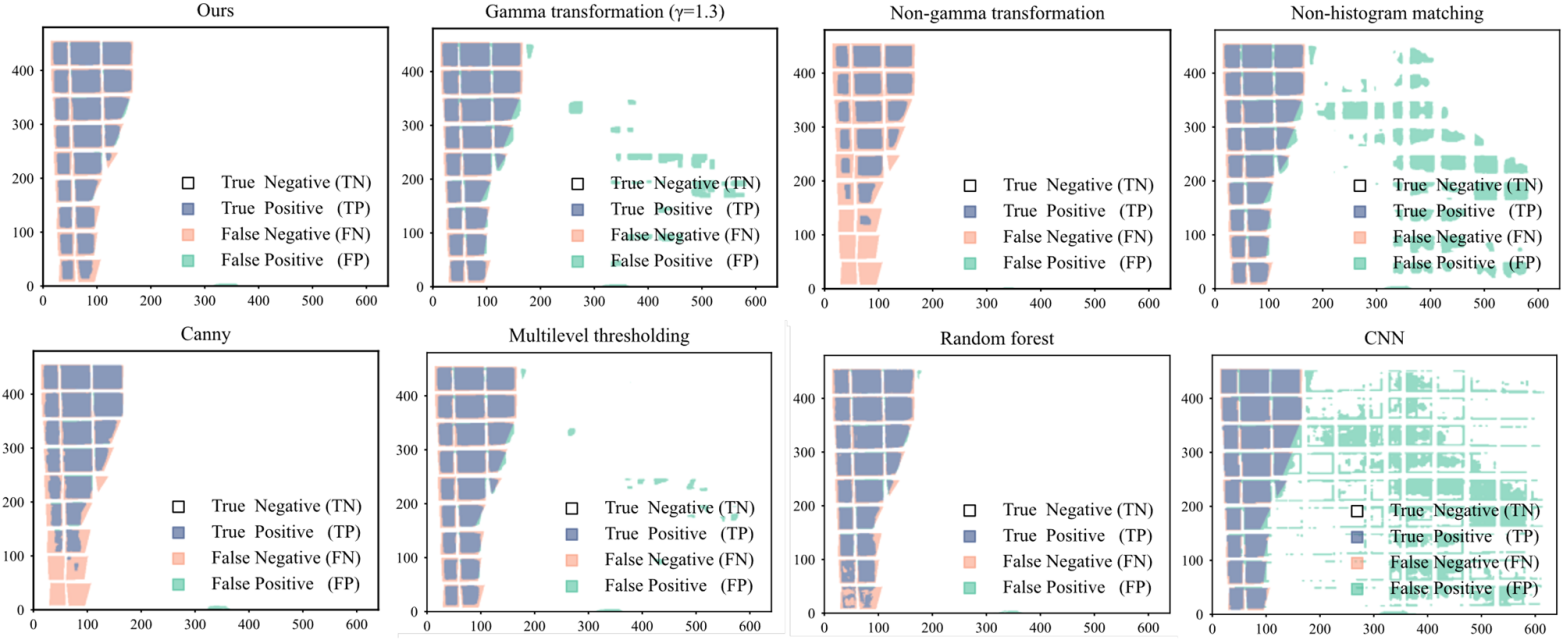
Comparison of five models for shadow segmentation.

The shadows detected by the model are first compared with the original image mask overlay to evaluate the performance of different shadow detection algorithms. As shown in Fig. 15, the detection performance of the eight models on local shadows is compared, where the white part represents TN, the pink part represents FN, and the green part represents FP, the blue part represents TP.

Comparing the detection results in Fig. 15, it can be seen that in terms of shadow detection accuracy and completeness, our method has the best detection performance for both shadows and nonshaded. This is followed by random forest and multi-threshold segmentation. The existing recognition methods based on canny edge detection and the method without gamma transform are incomplete for shadow recognition. In contrast, the comparison methods with γ = 1.3, no histogram matching, and CNN were not accurate for non-shadow detection.

**Fig. 16:**
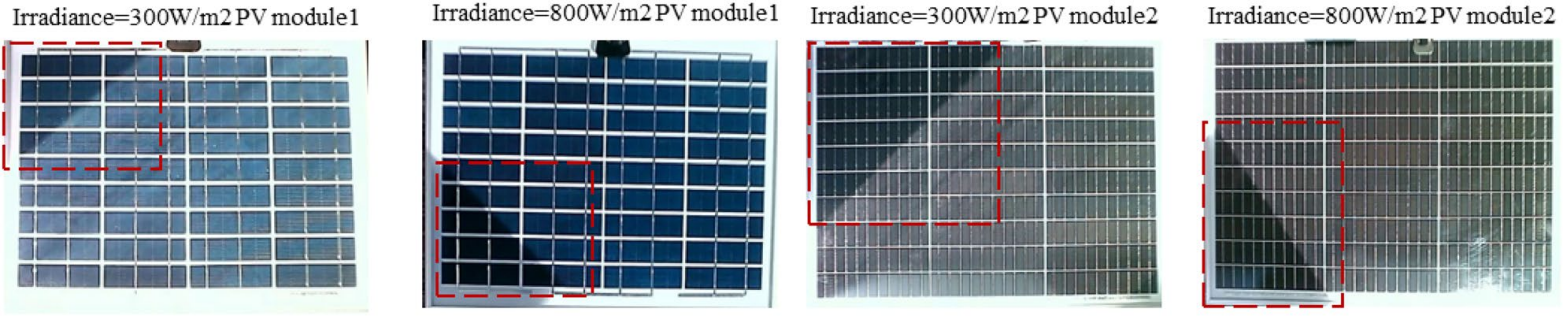
Comparison of five models for shadow segmentation.

This study further investigates the performance of the proposed method in shadow detection for different types of photovoltaic modules under different illumination conditions. Specifically, two types of PV modules are considered: PV module 1, which consists of 9 rows and 4 columns of deep blue silicon cells, and PV module 2, which consists of 10 rows and 3 columns of dark gray silicon cells. Figure 16 shows the images taken from these two types of PV modules under two different lighting conditions, namely a high light condition with an irradiance of 800W/m2 and a low light condition with an irradiance of 300 W/m2, resulting in a total of 4 scenarios. In these images, the areas surrounded by red boxes indicate the shadow areas.

**Fig. 17:**
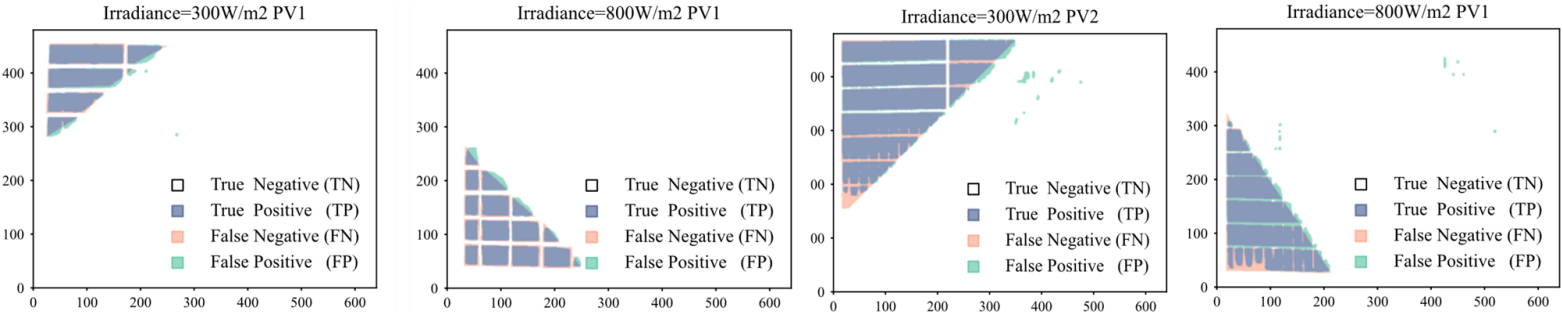
Comparison of five models for shadow segmentation.

Figure 17 shows the superimposed effect of the shadow truth values and the model recognition results for the above four cases, where the white part represents TN, the pink part represents FN, and the green part represents FP, the blue part represents TP. The above results show that the proposed method is capable of accurately recognizing the shaded and non-shaded areas of two different components under different lighting conditions. This provides an effective tool for shadow detection and identification, and helps to improve the accuracy and reliability of shadow detection. Overall, this method shows good performance in shadow detection and recognition and has a wide range of applications.

**Fig. 18:**
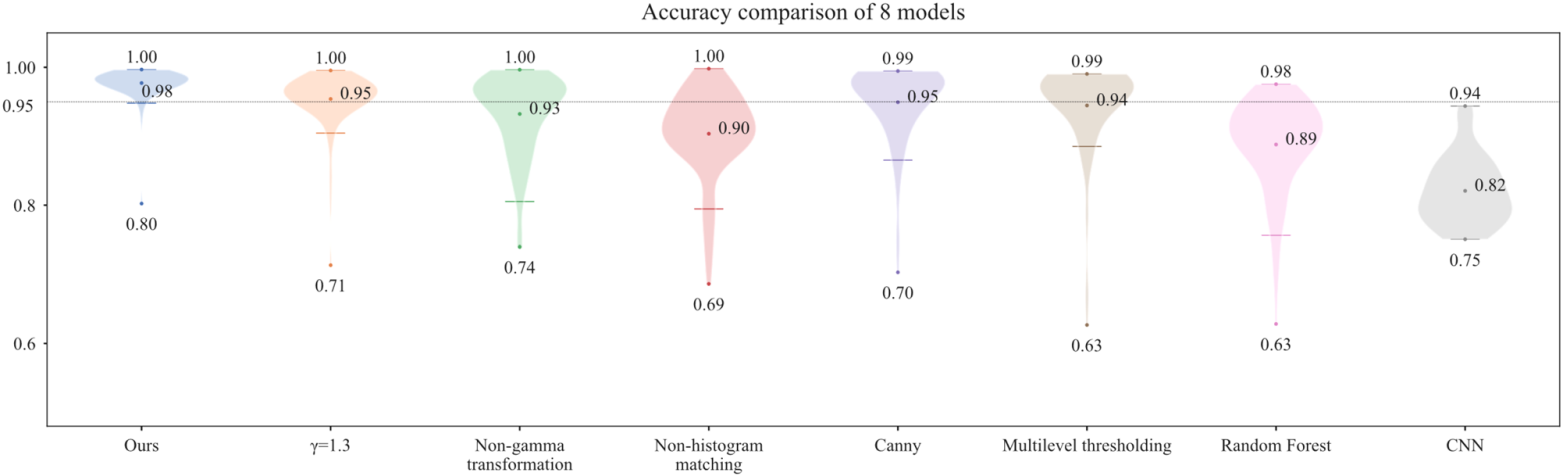
Evaluation of the accuracy of the eight models.

Figure 18 is the accuracy result that shows the detection performance of the eight groups of models from the video splits into 1000 frame images. The average ACC of the proposal model in this paper reaches 0.98, higher than the method based on the Canny operator. Even with the lowest accuracy, the designed method outperforms all the other groups, which shows that our method excels in robustness and accuracy. Furthermore, the control group of CNN has the lowest average accuracy among the five model groups, with an average value of 0.82.

**Fig. 19:**
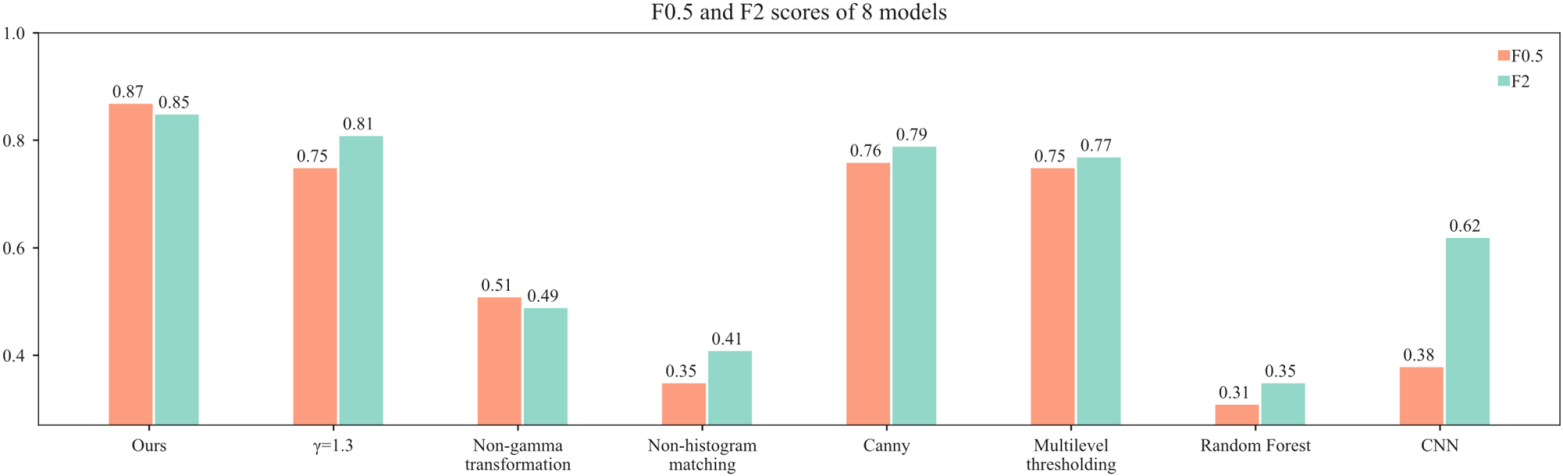
Comparison of F0.5 and F2 scores for eight models.

To further evaluate the performance of the eight models, F0.5 and F2 scores are calculated in this paper. As shown in Fig. 19, the F0.5 and F2 scores of the model in this paper are the highest, 0.87 and 0.85, respectively, indicating that the model has high recall and precision for positive examples. Random forest has the lowest F0.5 and F2 scores, 0.31 and 0.35, respectively, and the F2 score of the CNN model is much higher than the F0.5 score, indicating that the model has low precision and is inaccurate in recognizing the background.

**Fig. 20:**
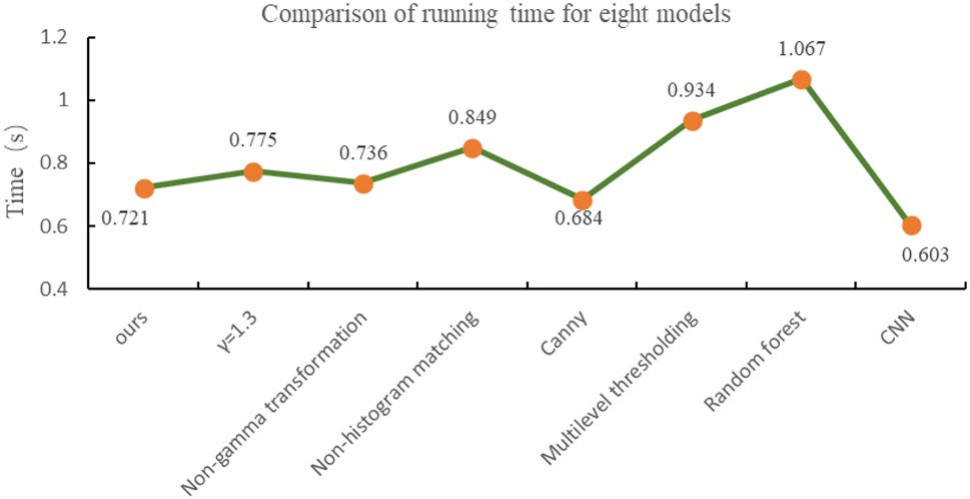
Comparison of running time for eight models.

To visualize the specific running time of the eight models, under the same running conditions, each model processes the image 100 times in hardware device as Table 2. As shown in Fig. 20, the average processing time of each model for one frame is plotted. Among them, the CNN model has the shortest running time of 0.603 s, the Random Forest model has the longest running time of 1.067 s, and the model in this paper has the middle ranking of 0.721 s.

**Fig. 21:**
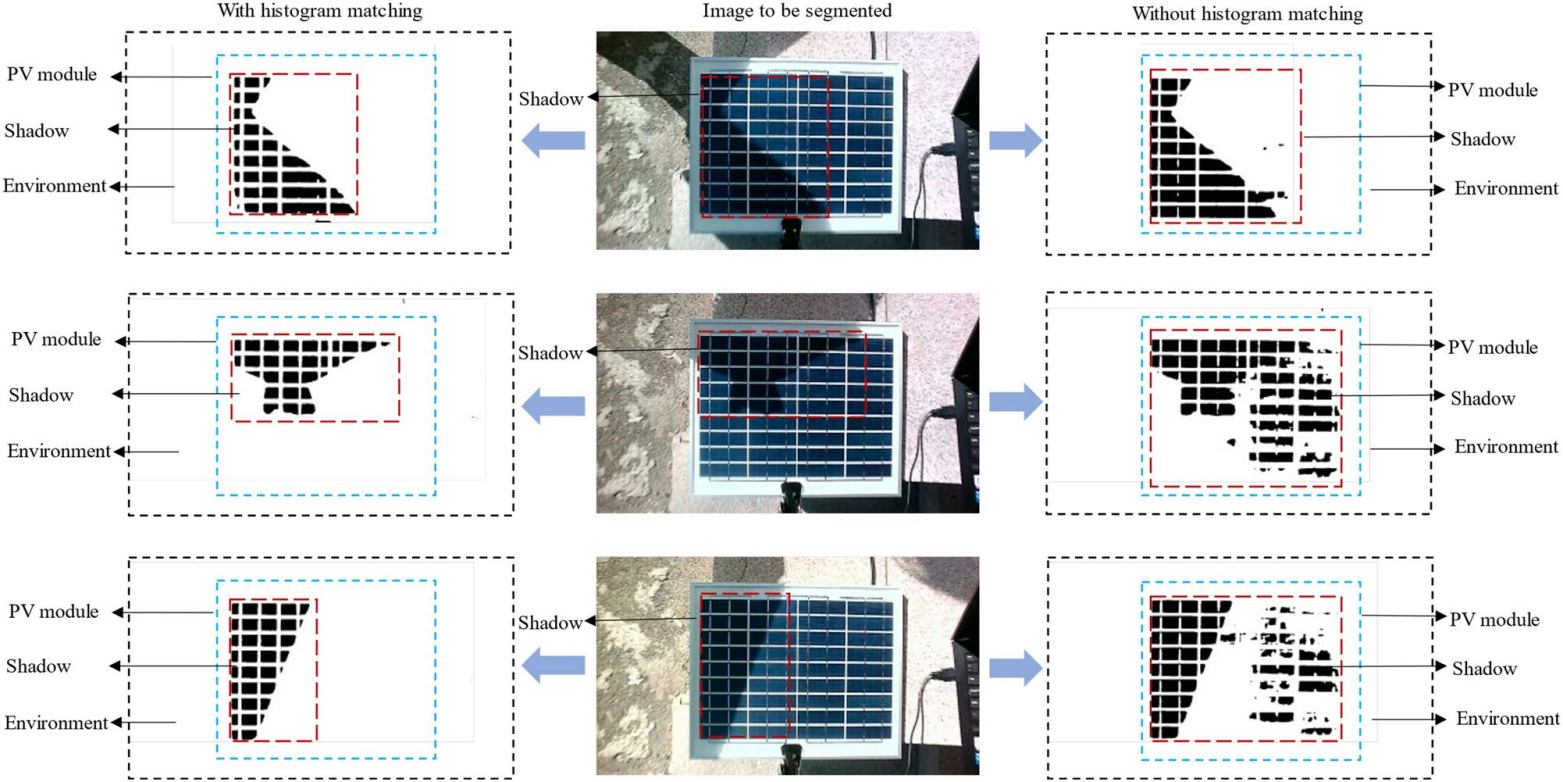
Comparison of shadow detection performance.

As shown in Fig. 21, shadow recognition performance of three sets is used to further evaluate the performance of this method. The left column of Fig. 21 is the effect of the method, i.e., adding histogram matching, the middle column is the original image to be recognized, and the right column is the control group. In the two groups of recognition results, black is the predicted shadow part, and white is the predicted nonshaded part. The blue dotted line region is the PV module, the surrounding information around the module is outside the region, and the red dotted line region is the detected shadow. It can be seen that the method in this paper works better than without histogram matching.

**Fig. 22:**
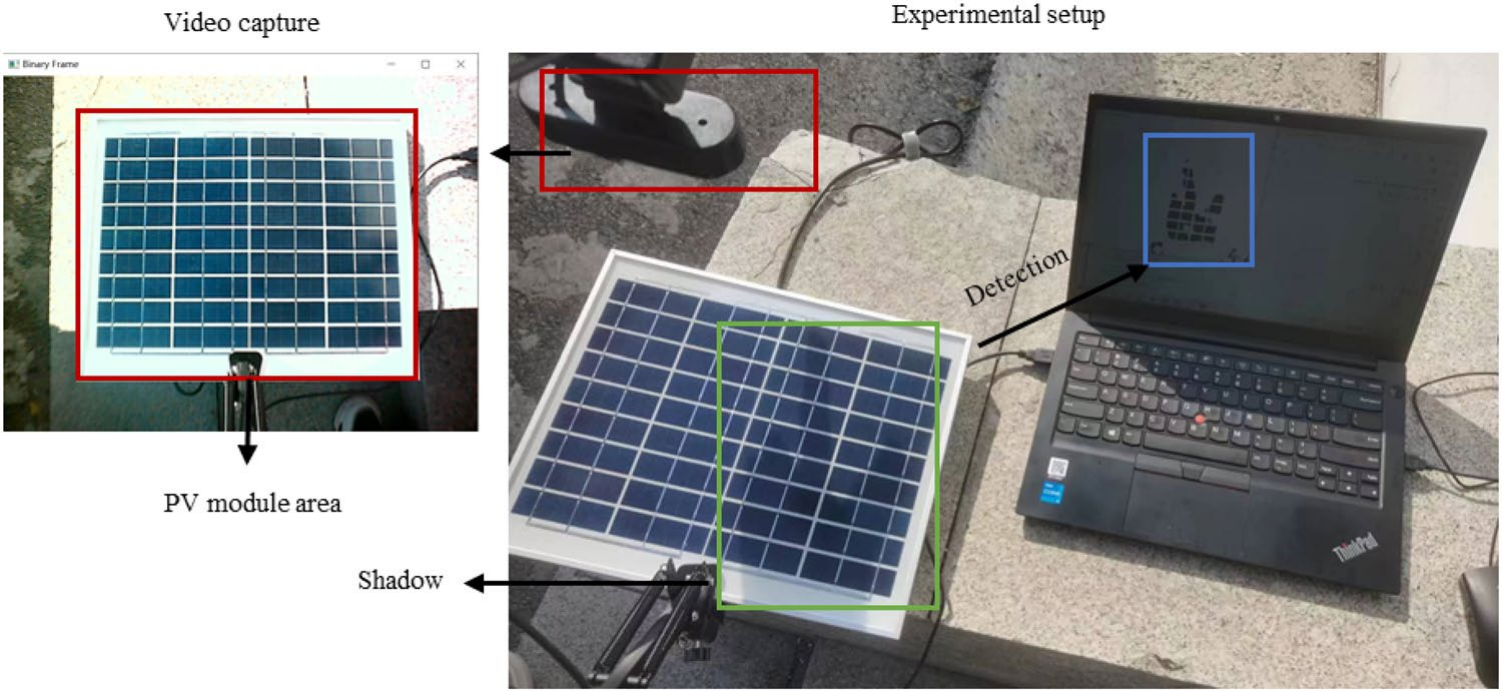
Real-time monitoring effect of the PV module shadow.

The practical application performance of the real-time monitoring method of PV module shadow based on histogram matching is shown in Fig. 20. The images of the PV module are captured by a fixed camera. The detection effect is displayed in real-time on the HMI of PC, with the shadowed areas shown as black and the nonshaded areas shown as white. The left image in Fig. 22 shows the captured image with the PV module in the red box. The right image in Fig. 22 shows the actual shadow area on the module in the green box and the detected shadow area in the blue box on the PC side.

In summary, the adaptive PV module shadow detection method by computer vision proposed in this paper performs well in completeness, accuracy, precision, recall rate, and practical application effect of shadow and nonshaded detection.

## Conclusions

This paper proposes a real-time PV module shadow monitoring method by computer vision based on histogram matching and gamma transformation. The method in this paper achieves state-of-the-art results in a real working environment.

In this paper, we first analyzed the histogram features of locally shaded PV modules, based on which coordinate constraints remove the environmental disturbance around the module, the gamut strength distribution is corrected by gamma transform, the image contrast is enhanced by using histogram matching, the shaded portion is preliminarily segmented by using gray-level slicing, and the closure operation and filter combination further refine the shadows. The average recognition ACC of this method is verified to be 0.98 by the test set, which is higher than the existing Canny edge detection recognition method. The method's F0.5 and F2 values are 0.87 and 0.85, respectively, which are good in terms of precision and recall. In addition, the average time required by the method to process an image frame is 0.721 s, which has good real-time performance. Furthermore, the real-time monitoring effect is verified by practical tests, which provide an excellent detection performance and high real-time performance even in complex working environments.

The limitations of this method are twofold:

Firstly, this study, as an application of a methodology, has empirically confirmed that the threshold of gray-level slicing is universal during the summer and autumn seasons at a specific geographical coordinate (45°44′26″N, 126°43′03″E). However, if the device is deployed during periods of significant light changes, initial validation is required to derive a threshold with higher adaptability.

Secondly, the method is exclusively specialized for individual identification of shadows on the surface of PV modules. If an object with a color similar to the shadow appears on the photovoltaic component, it may be misidentified as a shadow. However, in the actual working environment, the possibility of an object with a color similar to the shadow appearing on the surface of the PV component is extremely small. Furthermore, even if such a situation does occur, this object would produce the same effect as the shadow, that is, blocking the photovoltaic component and causing its power to decrease. Therefore, identifying it actually helps to improve the efficiency of the photovoltaic component.

Given the complexity and variability of situations that may encounter in the actual operation of PV modules, this study, as an application of a method, cannot fully verify all situations. It can be further optimized and improved in subsequent studies, mainly including the following aspects:

1. In terms of threshold selection, develop adaptive algorithms or use optimization algorithms to dynamically adjust the threshold according to real-time lighting conditions to improve shadow detection accuracy.
2. For the problem of false positives, the algorithm can be trained and optimized by combining the present method with CNN to improve its robustness in the real working environment.

The limitations of the present method allow an in-depth discussion of the above solutions. They also provide a starting point for future research.

We believe that our work is an important reference for realizing real-time shadow monitoring for large-scale PV arrays by providing solutions to some key details, and we look forward to furthering research on improving the model’s performance and generalizability.

## Data Availability

The datasets used and analysed during the current study available from the corresponding author, [Z.Q.], on reasonable request.
